# Brushed lubricant-impregnated surfaces (BLIS) for long-lasting high condensation heat transfer

**DOI:** 10.1038/s41598-020-59683-z

**Published:** 2020-02-19

**Authors:** Donghyun Seo, Jaehwan Shim, Choongyeop Lee, Youngsuk Nam

**Affiliations:** Department of Mechanical Engineering, Kyung Hee University, Yongin, 446-701 South Korea

**Keywords:** Mechanical engineering, Surfaces, interfaces and thin films

## Abstract

Recently, lubricant-impregnated surfaces (LIS) have emerged as a promising condenser surface by facilitating the removal of condensates from the surface. However, LIS has the critical limitation in that lubricant oil is depleted along with the removal of condensates. Such oil depletion is significantly aggravated under high condensation heat transfer. Here we propose a brushed LIS (BLIS) that can allow the application of LIS under high condensation heat transfer indefinitely by overcoming the previous oil depletion limit. In BLIS, a brush replenishes the depleted oil via physical contact with the rotational tube, while oil is continuously supplied to the brush by capillarity. In addition, BLIS helps enhance heat transfer performance with additional route to droplet removal by brush sweeping. By applying BLIS, we maintain the stable dropwise condensation mode for > 48 hours under high supersaturation levels along with up to 61% heat transfer enhancement compared to hydrophobic surfaces.

## Introduction

Condensers are indispensable components in a variety of energy applications, including power plant, thermal management, air conditioning, refrigeration, water desalination, distillation, cooling tower and water harvesting. The improvement in the heat transfer performance of the condensers can provide significant energy and cost savings to those systems^1–5^. As such, the development of advanced condensers for high heat transfer application has been a subject of great interest for decades^2,6,7^. Recently, lubricant-impregnated surfaces (LIS) have attracted much attention due to very high mobility^8–11^ of liquid droplets on LIS resulting from the extremely small contact angle hysteresis ( < 5°). A suitable lubricant infused into hydrophobic micro/nanoscale structures provides an ultra-smooth and water-repellent interface for water condensation, facilitating shedding of droplets along with rapid reactivation of nucleation sites compared to hydrophobic (HPo) surfaces^12–17^. In addition, the flat lubricant interface does not suffer flooding phenomenon that afflicts typical superhydrophobic surfaces at a relatively high supersaturation level^12,15,18^. Thanks to these advantages, LIS was reported to improve the heat flux by ~400% and ~30% over the conventional filmwise and dropwise condensation of hydrophilic and hydrophobic surfaces, respectively^16^.

However, despite a remarkable enhancement of condensation heat transfer on LIS, there have been concerns about the depletion of the lubricant oil, which is closely linked to the lifetime and/or durability of the surface. For example, a shear stress caused by the liquid flow or drop impact can displace the impregnated oil from the surface^19,20^. Also, due to the difference in the interfacial tensions between the lubricant oil and water, a thin oil layer wraps over the water droplet, while the oil ridge forms around the droplet^12,21,22^. This geometric configuration contributes to the concomitant oil loss when water droplets were removed from the surface^23^. Nevertheless, few studies have focused on oil depletion limit at the condensation condition, where numerous discrete droplets are continuously condensed and removed from the surface, giving rise to continuous oil drainage.

There have been several efforts to prevent and/or delay oil depletion based on passive approaches including structural design^24,25^, the tuning of lubricant viscosities^26,27^ and wettability patterning^28^. For example, a nanostructured surface was shown to better resist oil drainage by the liquid flow compared to a hierarchically structured surface^24^. Also, it was shown that a low viscosity oil was more advantageous for the lubricant retention against the external flow in microchannels due to smaller shear stress at the lubricant layer^26^. On the other hand, condensation conditions, including complex droplet growth, coalescence and shedding, showed better lubricant stability at higher oil viscosity^27^. Also, the wettability patterning, such that periodical hydrophilic regions were embedded on LIS, was shown to suppress the shear- and/or gravity-driven oil drainage^28^. However, the effectiveness of these passive methods were never tested under a harsh condensation environment involving high heat transfer. Furthermore, the previous passive means are mostly preventive measures, and would become obsolete after the oil depletion. Therefore, active methods, which can sustainably replenish or restore the lost oil under harsh condensation environment, need to be developed for practical applications of LIS, but the active approaches have not been reported yet to the best of the authors’ knowledge.

**Figure 1:**
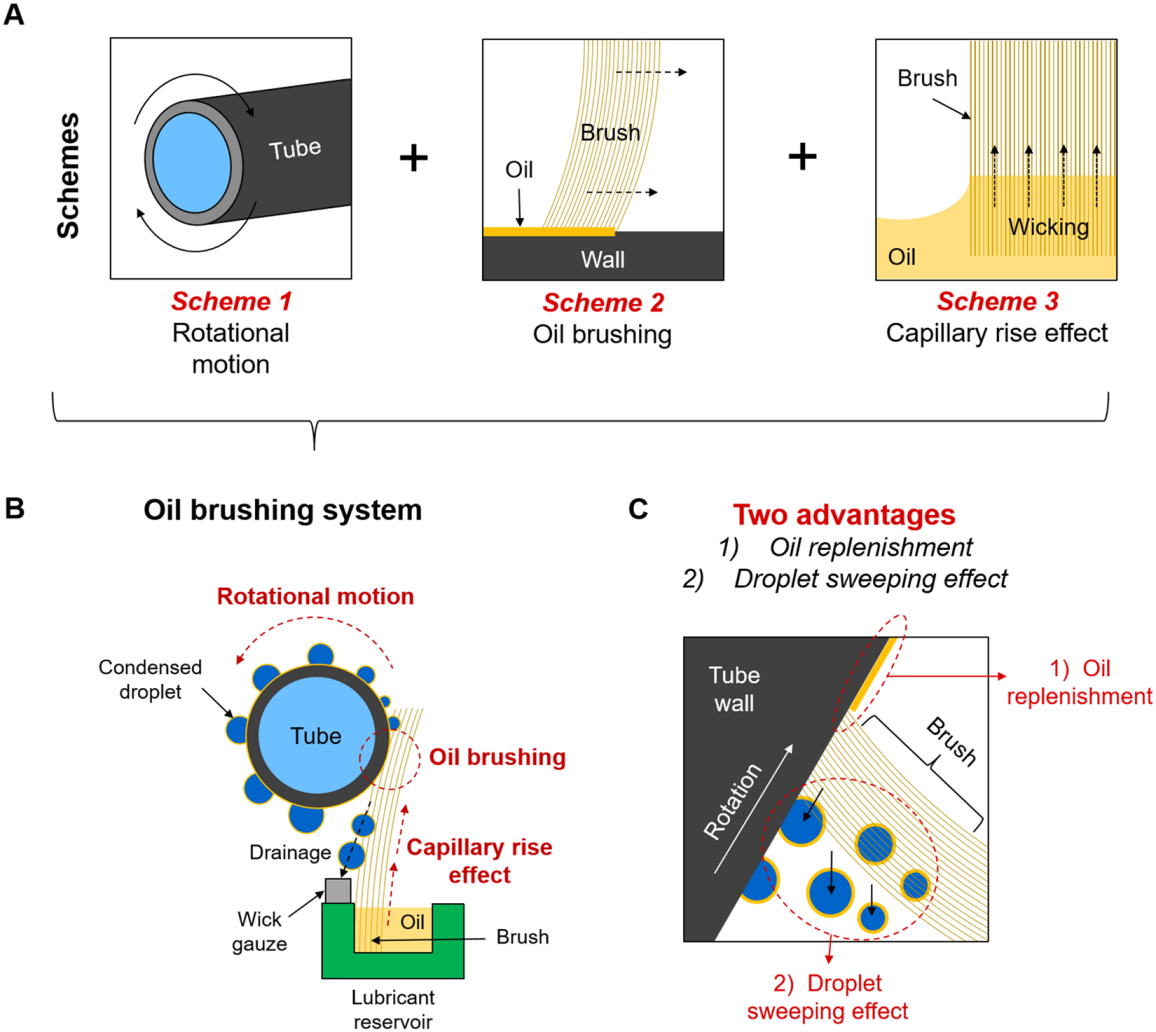
Concept of the brushing system. (**A**) Our brushing system utilizes the three schemes: the rotational motion of a tube (Scheme 1), the oil brushing (Scheme 2) and the capillary rise effect through the oil-wet brush (Scheme 3). (**B**) The system works with the following process. The rotational motion of the tube allows the stationary brush to thinly and continuously apply the lubricant layer onto the LIS tube wall. The oil at the brush hairs is spontaneously and quickly replenished from the oil reservoir by the principle of the capillary rise effect. At the same time, the brush sweeps condensing droplets sitting in the path of travel. The swept droplets fall along the brush and are drained out as soaking in a wick gauze. (**C**) The system provides two clear advantages: the oil replenishment and the droplet sweeping effect. These two advantages lead to durable and high condensation heat transfer performance at a wide range of supersaturation levels.

In this study, we propose an active oil replenishing system for tubular LIS condensers, which enable us to achieve remarkable improvement in both the surface durability and the condensation heat transfer coefficient at high supersaturation levels. Our approach, which is schematically shown in Fig. 1A,B, combine three schemes: the rotational motion of a tube (Scheme 1), oil brushing (Scheme 2) and oil refilling within brush by capillary rise (Scheme 3). The physical contact between the rotational tube wall and the soft brush hairs ensures lost oil on the condenser to be reliably replenished, while the oil within the brush hairs are spontaneously refilled from the oil reservoir by the capillary rise effect. Note that, in addition to the oil replenishment, BLIS can impart additional benefit to heat transfer performance by facilitating the droplet removal via brush sweeping (Fig. 1C). These two characteristics can provide the significant improvement in both the LIS durability and the condensation heat transfer over the conventional LIS at a wide range of supersaturation levels.

To evaluate the effectiveness of the proposed schemes, we first study condensation behaviors on the conventional LIS condenser (LIS), highlighting its oil depletion limit based on the temporal change of the sliding droplet size and ESEM (environmental scanning electron microscope) observations. After presenting the oil depletion stage over time, we quantify the longevity of LIS under various condensation rate conditions. Then, a long-term condensation performance of BLIS: the sliding droplet size and condensation heat transfer coefficient is presented along with those on other control surfaces with different wettabilities, including LIS, hydrophobic surface (HPo) and superhydrophobic surface (SHPo). Unlike other control surfaces, a long-term, stable dropwise condensation mode on BLIS is maintained up to 48 h without any sign of deterioration.

Then, the improved heat transfer performance on BLIS over other surfaces is explained based on additional droplet sweeping by brushing. Finally, using a well-controlled environmental chamber, we demonstrate that BLIS provides the highest condensation heat transfer coefficient (>72 kW/m^2^·K) at a wide range of supersaturation levels, achieving up to 61% heat transfer enhancement over hydrophobic surfaces.

## Results and Discussion

### Durability and condensation performance of conventional LIS condensers

**Figure 2:**
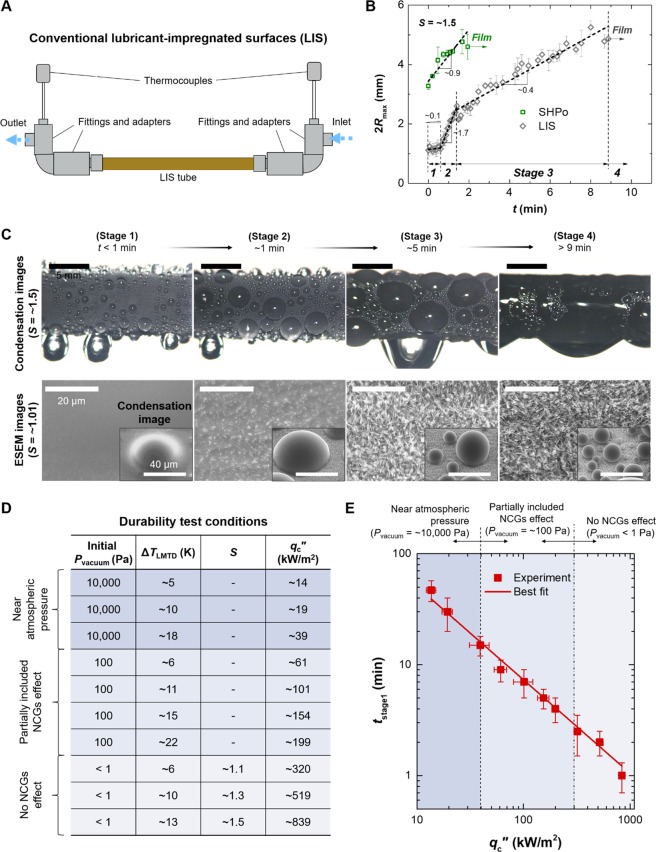
Durability and condensation performance of conventional LIS. (**A**) A conventional LIS condenser tube, symbolized as LIS, is connected to the flow line of cooling water to study the durability. Stainless steel fittings and adapters are used to supply cooling water inside the tube and to fix two thermocouple probes for measuring inlet and outlet temperatures. (**B**) Changes in 2*R*_max_, which is the maximum droplet diameter when a droplet begins to fall from the surface via gravity, as a function of time at *S* of ~1.5. LIS undergoes a large change in condensation behavior as impregnated oil is quickly depleted, showing 4 stages of the oil depletion depending on the slope. (**C**) Representative condensation images at *S* of ~1.5 and ESEM (environmental scanning electron microscope) images at *S* of ~1.01 on LIS of each stage. Small and mobile droplets are only found in stage 1, where the sufficient lubricant layer remains and the condensed droplets float on the oil layer. 2*R*_max_ markedly rises as the underlying nanostructures begin to appear above the oil-vapor interface due to the oil depletion (stage 2). Further loss of oil leads to the smooth increase in 2*R*_max_ (stage 3). When LIS is completely covered with water film, there remains no oil within the nanostructures (stage 4). (**D**) Test conditions to evaluate the durability of LIS. The non-condensable gases (NCGs) effect is carefully controlled by the initial vacuum pressure *P*_vaccum_. The condensation rate is quantified as the logarithmic mean temperature difference *T*_LMTD_, *S* and condensation heat flux *q*_c_″ which is calculated by multiplication of *T*_LMTD_ and the condensation heat transfer coefficient *h*_c_. (**E**) The durability is evaluated as *t*_stage1_ as a function of *q*_c_″. Here *t*_stage1_ indicates the maximum duration time that stage 1 can be sustained. The red symbols represent *t*_stage1_ experimentally obtained by tracing the change in 2*R*_max_, and the red line denotes the best linear fit. The *t*_stage1_ result displays that even under the low condensation rate conditions, LIS loses stage 1 within 1 hour, which implies that LIS is very vulnerable to condensation environment.

Figure 2A displays the schematic of the conventional LIS condenser tube (LIS) placed within the environmental chamber. Details regarding sample fabrication methods can be found in Methods. The LIS tube is connected to the flow line of cooling water consisting of stainless steel fittings and adapters. Two thermocouple probes are fixed at the flow line to measure inlet and outlet temperatures. Figure 2B shows the changes in 2*R*_max_, i.e., the maximum droplet diameter when a droplet begins to fall from the surface via gravity, on SHPo and LIS surfaces as a function of time at *S* = ~1.5. Here, the supersaturation level *S* is given as *P*_v_/*P*_sat_ (*T*_s_) where *P*_v_ is the vapor saturation pressure and *P*_sat_ (*T*_s_) is the saturation pressure at the tube surface temperature *T*_s_. Droplets on SHPo show the rapid increase in 2*R*_max_ with the slope of ~0.9, eventually reaching the complete transition into water film in less than 3 min due to the surface flooding phenomenon. Meanwhile, LIS shows a larger change in 2*R*_max_ with time, as oil depletion progresses. Based on the slope changes of 2*R*_max_, we define four different stages of oil depletion. Initially, the sliding droplet size on LIS is about 2*R*_max_ = ~1.2 mm, which is comparable with the sliding droplet size in previous studies^14,16^ (stage 1). Then, 2*R*_max_ markedly rises with the slope of ~1.7 (stage 2), followed by more gradual increase with the slope of ~0.4 (stage 3) until drop condensation mode transitions to film condensation mode (stage 4). It is generally known that smaller sliding droplet size provides better heat transfer performance^29^; hence, the increase in the droplet size on LIS implies degradation of the heat transfer performance over time.

To associate the observed droplet size with the oil depletion stage on LIS, we observe the condensation behaviors on each stage using CCD camera and ESEM (environmental scanning electron microscope), as shown in Fig. 2C. The condensation images using CCD camera are captured at *S* = ~1.5, while ESEM images are obtained at lower *S* = ~1.01 in order to capture clearer images. To ensure the identical oil depletion stage during ESEM measurement, we place the flat LIS sample inside the chamber at *S* = ~1.5 until the target oil depletion stage, and then lower the supersaturation level to *S = *~1.01 before ESEM measurement. In stage 1, the CuO nanostructures are completely submerged in oil. Hemispherical droplets grow within the oil layer, and their contact lines are not clearly visible due to the wetting ridge around the droplet. Oil layer above the nanostructures and small, mobile droplets on the surface confirm a typical LIS. Meanwhile, after stage 1, the underlying CuO nanostructures begin to be exposed as a result of the oil depletion. The water droplets directly in contact with the exposed nanostructures experience the larger pinning force due to the increased surface roughness, manifested by a larger 2*R*_max_ than stage 1. In stage 3, growing droplets exhibit almost spherical shape, as the bottom contact area beneath the droplets becomes much smaller than that in stage 2. As detailed in the Supplementary Note 2, when the thickness of the lubricant layer decreases below a critical thickness, the energy barrier for droplet growth above the nanostructures becomes smaller than that for spreading out through the nanostructures. Although this depletion stage normally decreases the pinning force acting on the droplet contact line, it leads to the higher 2*R*_max_ on LIS tube due to the flooding phenomenon at high *S* of ~1.5 as the lubricant layer is progressively replaced by water condensates instead of air (stage 3). Detailed discussion about the droplet wetting morphology and the critical thickness of the lubricant layer can be found in Supplementary Note 2. Finally, in stage 4, no lubricant layer is observed on LIS, showing the similar condensation behavior as that of SHPo under ESEM (stage 4). It is important to note that the different droplet morphologies in CCD and ESEM images in stage 4 can also be attributed to flooding phenomenon at higher *S*. Indeed, it has been shown that SAM coated CuO nanostructures exhibits the jumping condensation only at a small supersaturation level (*S* ≤ 1.15), and becomes flooded beyond that range^30^.

The above results demonstrate that the desirable dropwise condensation on LIS is only guaranteed at stage 1. Therefore, the durability for LIS can be quantified based on the duration of the stage 1 before the onset of the stage 2. According to Fig. 2B,C, the duration time for stage 1 can be found by tracing the changes in 2*R*_max_, where 2*R*_max_ of ~1.2 mm and the slope of ~0.1 were used as the criteria for stage 1. Then, we conduct the durability test at various condensation rate conditions, as summarized in Fig. 2D. *P*_vacuum_ and *T*_LMTD_ are widely controlled to achieve various condensation rate conditions, from low *q*_c_′′ under NCGs to high *q*_c_′′ without NCGs. Here, the logarithmic mean temperature difference ∆*T*_LMTD_ is defined as [*T*_v–in_ - *T*_v–out_]/[ln(*T*_v–in_/*T*_v–out_)], where *T*_v–in_ = *T*_v_ - *T*_in_ and *T*_v–out_ = *T*_v_ - *T*_out_. *T*_v_ is the vapor saturation temperature, *T*_in_ and *T*_out_ are the inlet and outlet temperatures, respectively. The condensation heat flux *q*_c_′′ is given as *h*_c_∆*T*_LMTD_, where *h*_c_ is the condensation heat transfer coefficient. It is important to note that even at the same *T*_LMTD_, the different *P*_vacuum_ leads to the substantial difference in the condensation rates, as shown in *q*_c_′′. At *P*_vacuum_ = ~10,000 Pa, *q*_c_′′ over than 100 kW/m^2^ cannot be reached even when *T*_LMTD_ is increased by more than 20 K due to the large temperature drop across the thick NCGs barrier near the water droplet interfaces. Meanwhile, at pure vapor conditions at *P*_vacuum_ < 1 Pa, the small *T*_LMTD_ = ~6 K can lead to a high *q*_c_′′ exceeding 300 kW/m^2^ as the additional temperature drop by NCGs is absent.

Figure 2E displays the duration time *t*_stage1_ for stage 1 as a function of *q*_c_′′. The experimental data, denoted as the symbols, show that even at low *q*_c_′′ = ~14 kW/m^2^, i.e., the minimum *q*_c_′′ in the present study, the stage 1 lasts less than 1 hour, indicating that the durability of LIS is highly compromised under the condensation environment. The solid line in the graph represents the best fit to the experimental data with the correlation of log_10_(*t*_stage1_) ∝ −log_10_(*q*_c_′′). This correlation shows that *t*_stage1_ is inversely proportional with *q*_c_′′; the lifetime for LIS is shortened as the condensation rate increases. The higher *q*_c_′′ means that more water vapors are condensed on the condenser surfaces along with a higher falling frequency of droplets. As the water droplets entrains the impregnated oil in the form of wrapping layers and wetting ridges^12,19–21,23^, the removal of droplets from LIS also results in the oil removal from the surface. Therefore, a higher removal rate of droplets at the high *q*_c_′′ would lead to a faster depletion of the lubricated oil, manifested by the decrease of *t*_stage1_.

In this section, we can draw the following conclusions about LIS. The lifetime and/or durability of LIS become problematic under the condensation environment, which is aggravated by physicochemical properties of impregnated oils on LIS. Particularly, the high condensation heat transfer on LIS is difficult to maintain due to accelerated oil depletion. Although passive approaches might help delay the oil depletion^24–28,31^, they are not the perfect solution as they cannot completely prevent the oil loss. Therefore, an active approach, which can reliably replenish the depleted oil on demand, is preferable to a passive approach. Here, we propose one active approach, the oil brushing system as shown in Fig. 1, which can overcome the oil depletion limit in the conventional LIS.

### Brushed lubricant-impregnated surfaces (BLIS)

**Figure 3:**
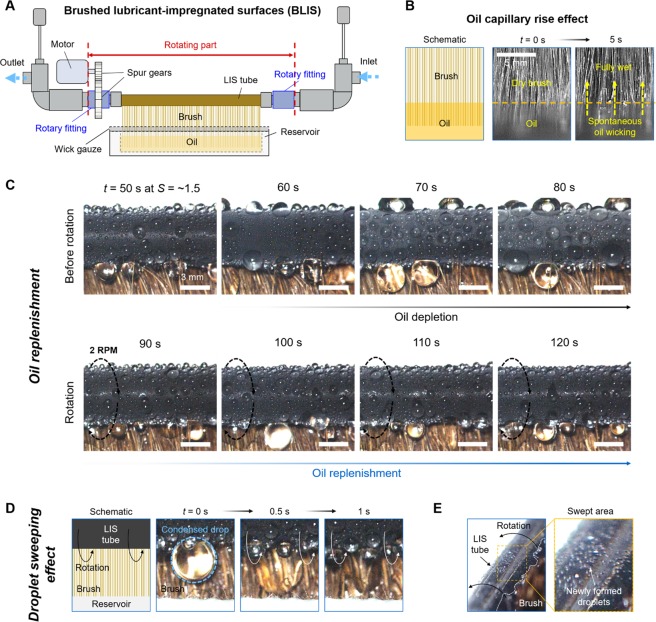
Brushed LIS (BLIS). (**A**) BLIS is realized by integrating a rotating tube, a DC motor, an oil reservoir and an oil-wet brush. The LIS tube is coupled with rotatable fittings which is integrated with the spur gears. The DC motor is connected to the spur gears to rotate the tube at a controlled RPM. The brush, which is partially submerged in oil in the reservoir, comes into contact with one side of the tube. (**B**) The oil-wet brush spontaneously and quickly sucks up the oil from the reservoir by the capillary rise effect. Such oil suction becomes the driving force that allows the oil in the brush to be continuously replenished from the reservoir. (**C**) Condensation images before and after the rotation of the tube. Before the rotation, BLIS undergoes rapid oil depletion and condensation performance degradation, like LIS. When the tube begins to rotate at 2 RPM, the lost oil is quickly replenished. (see Movie S1) (**D**,**E**) The rotational motion and the physical contact of the brush onto the tube wall continuously clears the large condensed droplets. This droplet sweeping effect can bring the benefit to heat transfer by promoting the re-nucleation and reducing average droplet size hanging on the surface.

To test the effectiveness of the active oil brushing system against the oil depletion, we use the modified experimental setup as shown in Fig. 3A. The oil brushing system consists of a rotating tube powered by a DC motor, an oil reservoir and an oil-wetted brush. The rotational motion in scheme 1 is enabled by the rotatable fittings and the small DC motor. The motor rotates the LIS tube attached to the rotatable fittings via spur gears at a controlled RPM. According to the specifications of our motor system (DC motor and motor speed controller, see Methods), the motor only consumes ~1.8 kWh per year to sustain 2 RPM of the tube. The oil brushing in scheme 2 is realized by keeping one end of the soft brush hairs in contact with the bottom of the tube. In scheme 3, the other end of the brush is placed within the oil reservoir, so that the brush can spontaneously wick oil upwards by capillary rise effect. Figure 3B displays that the prepared brush quickly absorbs the oil from the reservoir in less than 5 s. This capillary rise effect is aided by the high oil wettability to the brush surface as well as the small radius of curvature within the dense brush hairs. Detailed experimental setup for the brushing system and the information about the brush are presented in Supplementary Note 3. The robustness of the CuO nanostructures during the brushing is presented in Supplementary Note 4.

Figure 3C displays condensation images at *S = *~1.5 before and after the rotational motion of the tube. Before the tube rotation, the LIS tube undergoes the rapid oil depletion. When the tube begins to rotate at 2 RPM, it appears that the oil is successfully replenished, as small, mobile sliding droplets in stage 1 are once again observed. Oil replenishment is initiated as soon as the tube wall contacts the brush by the rotational motion, regardless of the pre-existing water droplets on the surface. The spontaneous replacement of impaled water within the nanostructures by oil layer is driven by hemiwicking behavior. Thermodynamically, the spontaneous oil spreading on the textured surface in water can be expected when cos*θ*_o/w_ > [(1 − *ϕ*)/(*r* − *ϕ*)], where *θ*_o/w_ is the intrinsic contact angle for a droplets on a solid surface in a water environment, *r* the roughness factor and *ϕ* the solid fraction^32^. In case of the CuO nanostructures used in this study, *r* is ~10.2 and *ϕ* is ~0.023; therefore, the critical angle for the hemiwicking is *θ*_c_ = 84°, which is larger than experimentally measured *θ*_o/w_ of 28.9 ± 2.6°, satisfying cos*θ*_o/w_ > cos*θ*_c_.

Note that the further increase in RPM of the tube causes over-application of oil, giving rise to the droplet packing^33–35^. The packing configuration of droplets occurs when the intermediate lubricant film between adjacent droplets interrupts the droplet coalescence, while it can deteriorate the condensation performance^34^. The over-application of oil induces a thick intermediate lubricant film between droplets, increasing the oil drainage time and consequently resulting in the droplet packing, as shown in Supplementary Note 5. In addition, RPM should be faster than the oil depletion rate to continuously replenish lost oil. The lowest possible RPM can be determined when considering the oil depletion rate at tested supersaturation ranges. As shown in Fig. 2E, RPM should be faster at least 1 / 40 s = ~1.5 RPM. Therefore, throughout the whole study, we fixed the rotational speed at 2 RPM which provided stable oil layer without any droplet packing issue. Details regarding the RPM effect can be found in Supplementary Note 5.

There is another advantage for the oil brushing system to the condensation performance, i.e., droplet sweeping effect, as shown in Fig. 3D,E. The physical contact of the brush with the tube wall sweeps condensed droplets away and renews the surface area for the nucleation of new droplets, while the rotational motion helps the large droplets over the whole area to be easily removed from the surface by brushing. One can expect that the condensation heat transfer will be improved by this effect, since the higher distribution density of smaller droplets is known to be beneficial to heat transfer^29^. The effect of droplet sweeping on heat transfer will be discussed in more detail below.

#### Oil replenishment effect

**Figure 4:**
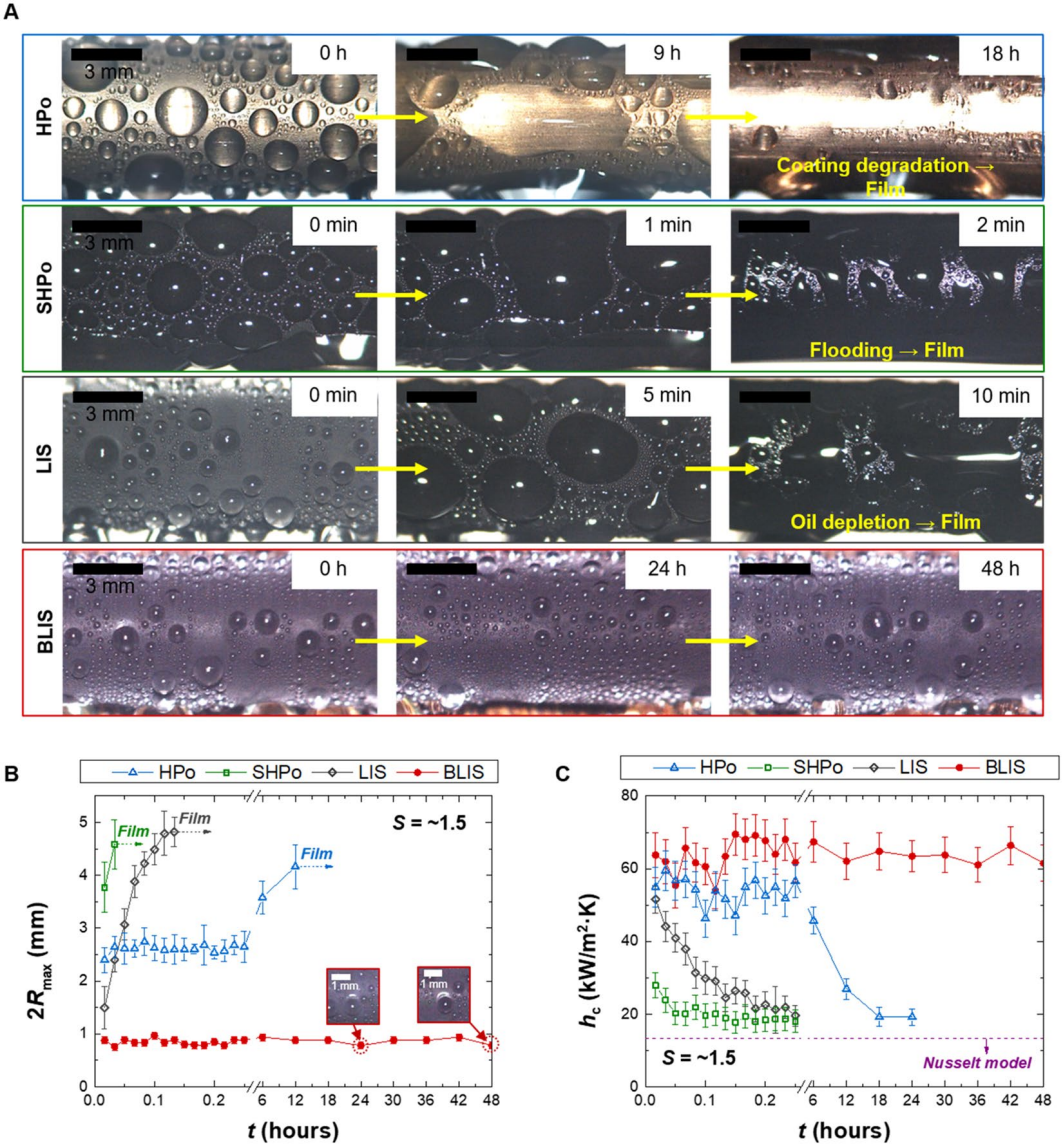
Oil replenishment effect of BLIS. (**A**) Condensation images of hydrophobic (HPo), superhydrophobic (SHPo), LIS and BLIS over time. All surfaces initially show dropwise condensation, but HPo, SHPo and LIS change into water film due to coating degradation, flooding and oil depletion problems, respectively. Meanwhile, BLIS maintains dropwise condensation for 48 hours at high *S* of ~1.5 because of the continuous oil replenishment. (see Movies S2‒5) (**B**,**C**) Changes in 2*R*_max_ and the condensation heat transfer coefficient *h*_c_ at *S* of ~1.5 over time. 2*R*_max_ of HPo, SHPo and LIS quickly rises until droplets become a water film. *h*_c_ for HPo, SHPo and LIS quickly reduces and approaches to the Nusselt model line since the water film provides large conduction resistance to heat transfer. Meanwhile, BLIS sustains the smallest 2*R*_max_ and the highest *h*_c_ regardless of time.

We evaluate whether the oil replenishment can help sustain dropwise condensation mode on BLIS without the oil depletion issue. To quantify the durability of BLIS, the long-term dropwise condensation behavior on BLIS is compared with other dropwise condensing surfaces including HPo, SHPo and LIS. Figure 4A compares condensation images of HPo, SHPo, LIS and BLIS over time. All surfaces initially show dropwise condensation; however, HPo, SHPo and LIS undergo the gradual change in condensation mode from dropwise condensation to water film condensation due to coating degradation (HPo), flooding (SHPo) or oil depletion (LIS). The ultra-thin hydrophobic coating layer cannot sustain long-term dropwise condensation at high supersaturation levels, as shearing by droplet movement and water penetration near the contact line gradually degrade the coating quality^18,36–40^. The presence of nanostructures on SHPo accelerates the transition into the filmwise condensation since the random nucleation within the nanostructures leads to irreversible surface flooding at a relatively high supersaturation level^18,30,41,42^. LIS also suffers flooding after underlying nanostructures are exposed after the complete oil depletion, as discussed in the previous section. Stable and long-term dropwise condensation is only observed on BLIS. BLIS maintains stable dropwise condensation up to 48 hours without any indication of surface degradation thanks to the continuous oil replenishment effect. The high durability of BLIS can be quantitatively verified by measuring the temporal changes in 2*R*_max_ and the condensation heat transfer coefficient *h*_c_ over time at *S = *~1.5, as shown in Fig. 4B,C. 2*R*_max_ for HPo, SHPo and LIS markedly increases and finally reaches the maximum value before the transition into the water film. During this transition process, *h*_c_ for the surfaces also quickly decreases and approaches to Nusselt model for filmwise condensation accounting for the increased conduction resistance across the water film. Meanwhile, BLIS shows nearly invariant 2*R*_max_ and *h*_c_ due to the successful oil replenishment.

The oil replenishment effect is maintained as long as the oil remains in the reservoir, which means that the amount of the oil in the reservoir determines the lifetime for BLIS. We can predict the minimum amount of oil required for sustaining BLIS by using the correlation between *t*_stage1_ and *q*_c_′′. From the fitting line in Fig. 2E, we obtain the following correlation, log_10_(*t*_stage1_) = 2.56–0.85log_10_(*q*_c_′′). Based on *t*_stage1_ at the different *q*_c_′′, we deduce the depletion rate of the oil volume at stage 1 such as *v*_o_ = *V*_o_/*t*_stage1_, where *V*_o_ is the volume of the impregnated oil at stage 1. The *V*_o_ is given by *V*_o_ = *A*_o_*δ*_o.stage1_, where *A*_O.D._ is the tube outer surface area and *δ*_o.stage1_ is the thickness of oil covering the surface at stage 1. Since stage 1 transitions to stage 2 when the underlying nanostructures begin to be exposed above the lubricant interface, as discussed in the previous section, we can estimate *δ*_o.stage1_ as *δ*_o.stage1_ = *δ*_o_ - *h*, where *δ*_o_ is the total thickness of the impregnated oil on the surface and *h* is the height of the underlying CuO nanostructures (*h* = ~1 μm). *δ*_o_ is measured by the difference in the mass of the tube before and after oil impregnation (*δ*_o_ is ~1.3 μm in this study). Details about the *δ*_o_ measurement are presented in Methods. This calculation predicts that under the present test tube dimension, the amount of the oil loss at *q*_c_′′ of 839 kW/m^2^ is about ~9.9 × 10^−12^ m^3^/s, which translates to ~0.58 kg per a year (density *ρ*_Krytox_ for Krytox is ~1.86 × 10^3^ kg/m^3^). If condensers are operating in a lower *q*_c_′′ ( = 39 kW/m^2^) in the presence of NCGs, BLIS could work for a year just with ~18 kg/m^2^.

#### Droplet sweeping effect

In addition to the significant improvement in the lifetime of oil lubrication layer, one sees that condensation heat transfer performance is enhanced on BLIS due to the droplet sweeping effect as shown in Fig. 4B,C. BLIS displays the smallest 2*R*_max_ and highest *h*_c_ among tested surfaces. Even compared to LIS at stage 1 (*t* < 1 min), BLIS demonstrates smaller 2*R*_max_ as well as higher heat transfer performance. A superior condensation performance of BLIS indicates that the brushing system provides additional benefit to the heat transfer enhancement in addition to the durability of oil lubrication layer.

**Figure 5:**
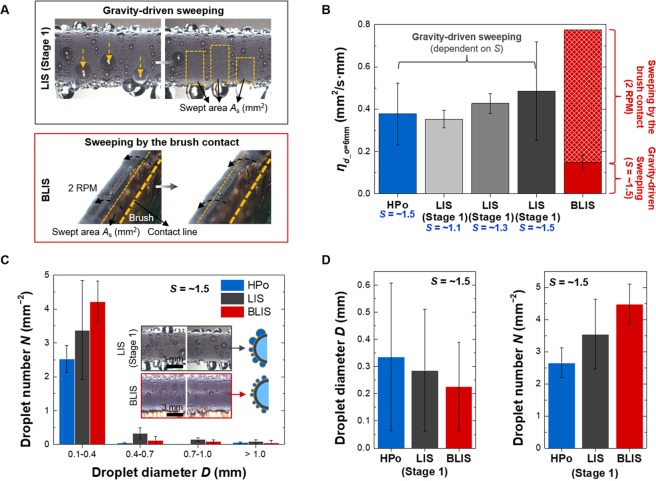
Droplet sweeping effect of BLIS. (**A**) Typical water-repellent surfaces clear their surfaces using the droplet sweeping by gravity, while BLIS utilizes not only the gravity-driven sweeping but also the physical contact of the brush. (**B**) The sweeping rate *η*_*d_o*=6mm_ for the tube with outer diameter *d*_o_ = 6 mm. *η*_*d_o*=6mm_ represents the swept area per unit time and length. BLIS shows the highest *η*_*d_o*=6mm_ compared to HPo and LIS, and such superior ability is attributed to the continuous sweeping by the physical contact of the brush. (**C**) Droplet number *N* and diameter *D* for droplets with *D* > 0.1 mm (0 ≤ *t* ≤ 60 at *S* of ~1.5). As shown in the inset images, LIS (stage 1) shows large droplets on the top and bottom of the tube wall due to the change in the downward force depending on the inclination angle of the surface. Meanwhile, BLIS continuously maintains the population of small droplets because of the sweeping effect attributed to not only the rotational motion but also the physical contact of the brush. As this result, BLIS shows a larger number of small droplets with *D < *0.4 mm than LIS (stage 1). (**D**) Averaged *D* and *N* also displays that BLIS has the smaller droplet size and larger droplet number compared to LIS, which implies BLIS has better condensation performance.

The droplet sweeping effect by the brushing system is quantitatively evaluated by the sweeping rate *η*_*d_o*=6mm_, shown in Fig. 5A,B. Note that the higher heat transfer performance associated with dropwise condensation is attributed to the facilitated droplet shedding accompanied by more rapid clearing of nucleation sites^29^. On typical surfaces with dropwise condensation, the surface is normally swept by sliding droplets driven by gravity, while on BLIS both gravity-driven droplets and brushing contribute to the surface sweeping, as shown in Fig. 5A. We denote the swept area per unit time and length for tube outer diameter of *d*_o_ = 6 mm as *η*_*d_o*=6mm_, and present the measured results in Fig. 5B. For HPo and LIS, *η*_*d_o*=6mm_ measured from the front of the tube is multiplied by two to account for the backside of the tube. In the case of BLIS, *η*_*d_o*=6mm_ by the gravity-driven sweeping is considered only from the value measured in the front and the additional *η*_*d_o*=6mm_ by the brush contact, named as brush sweeping, is added as *η*_*d_o*=6mm_ = 2 RPM × 1/60 s × *A*_o_ × 1 mm. The result of Fig. 5B displays that BLIS has the highest *η*_*d_o*=6mm_ among tested surfaces. The physical contact by the brush makes up the dominant portion of *η*_*d_o*=6mm_ for BLIS, demonstrating that the brushing system enhances the droplet sweeping.

The droplet sweeping effect is further investigated by measuring the droplet size distribution on the surface. Figure 5C,D display droplet number *N* and diameter *D* for droplets with *D* > 0.1 mm at 0 ≤ *t* ≤ 60 of *S* = ~1.5. Note that the droplets smaller than 0.1 mm could not be resolved due to the resolution limit of our CCD camera. As shown in the inset images of Fig. 5C, small droplets slide over the front side of the LIS tube, while relatively large droplets are observed on the top and bottom sides of the tube. Such large droplets are expected on the stationary tubular wall, since the gravitational force, the driving force for the droplet removal by sliding, varies with the surface orientation of the tube, such that it becomes smallest on the top and bottom sides of the tube. For example, the gravitational force for the droplet sliding on the inclined surface is given by *F*_g_ = *ρ*_w_*gV*sin*α*, where *ρ*_w_ is the water density, *g* is the gravitational acceleration, *V* is the droplet volume and *α* is the surface inclination angle. When *α* approaches to 0° or 180°, *F*_g_ for the droplet sliding also approaches to zero. The effect of surface orientation reduces the removal frequency of the droplets and increases the number of the relatively larger droplets, both of which are detrimental to heat transfer. Meanwhile, both the rotational motion of tube and the brush sweeping effect can significantly minimize the influence of surface orientation on the droplet size distribution. The droplets formed on the upper side of the tube can be easily removed without pinning, as *α* is continuously varied by the rotational movement. The droplets formed on the bottom of the tube are physically swept away by the brush, clearing the surface area for the growth of new droplets. Such a droplet growth process governed by droplet sweeping and renewal results in the higher *N* for smaller droplets in the droplet distribution, as shown in Fig. 5C. Averaged *D* and *N* in Fig. 5D also display the smaller *D* and the larger *N* on BLIS compared to HPo and LIS in stage 1. The increased number of smaller droplets implies a smaller heat conduction resistance on the surface or a higher heat transfer^18,43^, which demonstrates that the brushing system provides a droplet distribution favorable to heat transfer enhancement.

### Heat transfer performance evaluation

**Figure 6:**
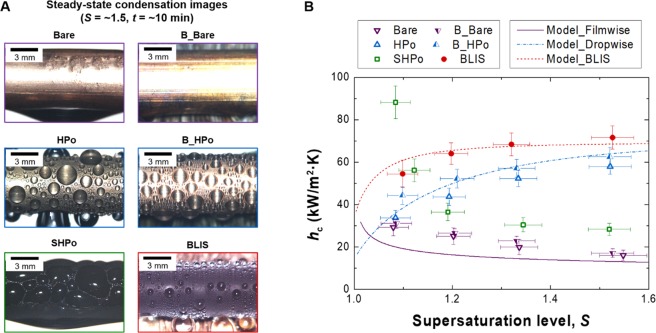
Condensation heat transfer coefficient of BLIS. (**A**) Steady-state condensation images of untreated bare (Bare), B_Bare, HPo, B_HPo, SHPo and BLIS at *S* = ~1.5. Here, B_Bare and B_HPo indicate brushed Bare and HPo using the 2 RPM brushing without the oil lubricant. The images are captured ~10 min after the condensation begins. Bare, B_Bare and flooded SHPo undergo filmwise condensation, while HPo, B_HPo and BLIS sustain dropwise condensation. Among the dropwise condensing surfaces, BLIS shows the smallest and the most mobile droplets due to the slippery characteristics and the droplet sweeping effect. (**B**) *h*_c_ as a function of *S*. Symbols represent experimental data and lines display prediction values obtained by the heat transfer model. Except for *h*_c_ of SHPo at *S* of ~1.1 in which the jumping condensation occurs, BLIS provides the highest heat transfer coefficient at a wide range of supersaturation levels. Droplet removal size $2\hat{R}$ corresponding to each data is presented in Table S1 of Supplementary Note 8.

The heat transfer enhancement by the droplet sweeping effect is directly evaluated by measuring *h*_c_ at various condensation conditions. Note that the precise measurement of the condensation heat transfer is possible only when NCGs are sufficiently removed from the environment, i.e. *P*_vacuum_ < 1 Pa^30^. In this pure vapor environment, high *q*_c_′′ more than 320 kW/m^2^ can be obtained, but the heat transfer coefficient of LIS at such a high *q*_c_′′ cannot be reliably measured because of too fast oil depletion (*t*_stage1_ < 3 min). Therefore, the heat transfer of BLIS is only compared with Bare (untreated hydrophilic), HPo and SHPo with their water contact angles shown in Methods. Figure 6A displays representative condensation images of the tested surfaces at *S = *~1.5. Figure 6B shows *h*_c_ as a function of *S*. Here, the symbols represent the experimentally measured data and the lines denote the prediction values estimated from the heat transfer model. Detailed calculation for *h*_c_ is shown in the Supplementary Note 6. The heat transfer data shows that BLIS has the highest heat transfer coefficient at a wide range of supersaturation levels, e.g., *h*_c_ for BLIS is 86‒346% and 26‒61% higher than Bare and HPo at the tested supersaturation ranges (1.1 < *S* < 1.6), respectively. Even though SHPo displays the highest *h*_c_ at *S = *~1.1 by the coalescence-induced droplet jumping, *h*_c_ dramatically decreases below the value for BLIS as a higher *S* due to the surface flooding limit^18,30,41,42^. As such, BLIS shows 75‒153% higher *h*_c_ than the flooded SHPo at 1.1 < *S* < 1.6. B_Bare and B_HPo are also tested to understand the droplet sweeping effect on *h*_c_. Here, B_Bare and B_HPo indicate brushed Bare and HPo using the 2 RPM brushing without the oil lubricant. The brushing helps to reduce the thickness of the water film on B_Bare, improving *h*_c_ by ~8%. In the case of B_HPo, the sweeping by the brushing promotes the formation of newly droplets on the surface, increasing *h*_c_ by ~17%. The results imply that the brushing is more effective for dropwise condensation, and the high *h*_c_ for BLIS will also be resulted from the droplet sweeping effect.

To understand the heat transfer results, we investigate the heat transfer model that incorporates the thermal resistance model for droplet growth, the emergent droplet wetting morphology, and droplet distribution theory^16,18,44,45^. Detailed calculations for the heat transfer model are shown in the Supplementary Note 7. We obtain the prediction line for BLIS in good agreement with the experimental data by using experimentally determined the droplet removal diameter $2\hat{R}$ of ~0.9 mm and contact angles (see Methods). $2\hat{R}$ for each surface according to the supersaturation level is presented in Table S1. The nucleation density *n* for BLIS is determined on the basis of the best fit between the analytical model and the experimental data. The predicted *n* for BLIS is achieved by assuming *n*_BLIS_ = 3 × *n*_HPo_, which means that the stable lubricant interface and ~2 times higher *η*_*L*=120mm_ by the droplet sweeping effect (Fig. 5B) provide ~3 times higher *n* on BLIS compared to HPo. *h*_c_ for Bare and flooded SHPo follows the Nusselt model due to the water film formation during condensation. This filmwise condensation provides much smaller *h*_c_ compared to dropwise condensation due to the water film acting as the dominant conduction resistance. The higher *h*_c_ for BLIS than that for HPo is attributed to the smaller $2\hat{R}$ and the higher *n* resulting from the enhanced surface sweeping effect (note that $2\hat{R}$ for HPo is ~2.7 mm). The smaller $2\hat{R}$ and higher *n* result in a higher distribution of smaller droplets, which helps reduce the overall conduction resistance on the surface and promote droplet growth, both of which lead to the increase in heat transfer rate.

## Discussion

From an industrial perspective, the addition of the brush and the rotating system for BLIS may increase manufacturing and maintenance costs. However, BLIS can provide not only the long-lasting dropwise condensation even at high supersaturations but also up to 4.5 times higher *h*_c_ compared to the filmwise condensation on Bare, which indicates that the brushing concept is worth considering in the real industry despite the extra costs. Here, we suggest several concepts and applications for utilizing BLIS. The proposed BLIS requires several essential components, including brush and rotating systems; hence, the concept may be more suitable for small to medium-scale condensers, which are relatively easy to install and maintain, such as small-scale chillers, thermal desalination systems of wastewater, and etc. Shell and tube condensers the types commonly used in these industrial applications, and the proposed concept can be implemented by replacing a tube at the center of the hexagonal bundle to a brush. As shown in Fig. S7A, the rotating brush placed at the center of the hexagonal bundle can continuously replenish oil to the stationary tubes placed at each vertex. In this case, wick channels in the vertical direction on the tube surface can be formed so that the surface not in contact with the brush can be also replenished with oil. Also, by rotating tubes, the stationary brush can replenish oil. In addition to the high supersaturation applications, BLIS can be utilized in fin-tube heat exchangers for dehumidifier and HVAC which are operated under relatively low *S*. As shown in Fig. S7B, the brush can be connected to a system that moves up and down linearly so that lost oil can be replenished and condensing droplets can be swept. The proposed oil supply concept can also be utilized in other applications, such as fog harvesting and anti-freezing. LIS has been well known to be a good fog harvesting and anti-freezing surfaces^15,34,46–48^. BLIS can be incorporated into fog harvesting devices by using the fog wind as the source to rotate the brush or the fog collector surfaces. In a refrigerator, evaporator coils, which usually suffer from freezing, can be incorporated with the brushing system to delay freezing and remove frozen droplets by the physical contact of the moving brush.

In summary, we demonstrated that BLIS can achieve the remarkable improvement in the durability of LIS by reliably replenishing the depleted oil during condensation, while providing additional benefit to the condensation heat transfer coefficient by brush sweeping. The traditional LIS suffered the unavoidable oil depletion limit during condensation, while undergoing the rapid degradation in the condensation heat transfer performance. Even typical dropwise condensing surfaces such as HPo and SHPo have showed critical limitations in sustaining long-term high heat transfer performance at high supersaturation levels due to the coating degradation and surface flooding. BLIS overcomes this critical limit by incorporating the active oil brushing system, which can provide highly durable, superior heat transfer performance at a wide range of supersaturation levels. We believe that BLIS can be a promising candidate for advanced condenser surfaces of various applications operating at a wide range of supersaturation levels, such as air conditioning, water collection, desalination, distillation towers, heat pipes, and power generation. In addition, we expect that the proposed oil brushing concept can contribute a wide variety of other applications utilizing LIS, such as anti-fouling, anti-corrosion, anti-freezing and self-cleaning, as providing the long-term durable lubricant interface.

## Methods

### Sample fabrication

We use commercially available copper tubes (99.9% purity) with outer diameter, *d*_o_ = 6 mm, inner diameter *d*_i_ = 4 mm and length *L* = 125 mm. During all chemical fabrication, the tubes are capped by nipples to prevent any functionalization of their inside. Once capped, the samples are cleaned in an ultrasonic bath with acetone and ethanol for 5 min, respectively and rinsed with de-ionized (DI) water at room temperature. Then the tubes are dipped into a 2.0 M hydrochloric acid solution for 10 min to eliminate any native oxide layers on the surface. Once complete, the tubes are thoroughly rinsed with DI water and then are dried with clean N_2_ gas. The CuO nanostructures are fabricated by immersing the cleaned tube inside a hot alkaline solution of ~95 °C composed of NaClO_2_, NaOH, Na_3_PO_4_·12H_2_O, and DI water (3.75:5:10:100 wt.%) for 15 min^18,49^. Sharp and knife-like CuO oxide structures fabricated by this method have height *h* of 1.0 ± 0.30 μm, pitch *p* of 300 ± 17 nm, solid fraction *ϕ* of ~0.023 and roughness factor *r* of ~10.2. After the oxidation process, the CuO nanostructured tubes are thoroughly rinsed with ethanol and DI water and are dried with clean nitrogen gas.

To render the surface hydrophobic, the samples are coated with self-assembled monolayers (SAMs)^18^. The substrates are treated by oxygen plasma to create hydroxyl group on the surfaces, and then they are dipped into a 1.0 wt.% hexane solution of HTMS (1 H,1 H,2 H,2 H-Perfluorodecyltrimethoxysilane, Sigma-Aldrich) for 2 h at room temperature. After the SAMs coating processes, the samples are strictly rinsed with ethanol and DI water and then sonicated in hexane for 5 min to remove excess polymerized monolayers on the surfaces. As the conventional LIS, the functionalized nanostructures are fully impregnated with the fluorinated oil of Krytox 1506 (oil-vapor interfacial energy *γ*_ov_ of ~17 mJ/m^2^ at 20 °C and kinematic viscosity of ~62 mm^2^/s at 20 °C). SEM (scanning electron microscope) images of the fabricated CuO nanostructures can be found in the Supplementary Note 10. We chose the Krytox 1506 as the lubricant oil for LIS since it has a very small vapor pressure *P*_v,Krytox_ of < 10^−12^ mmHg, which allows us to test LIS in our vacuum-based condensation chamber where *P*_vacuum_ is lowered down to < 1 Pa. In addition, Krytox satisfies the essential thermodynamic requirements for forming a stable and water-repellent lubricant interface on the nanostructured surface^8,12,22^.

The smooth hydrophilic (Bare) tube for the filmwise condensation is achieved by the oxygen plasma treatment of the cleaned tube for 10 min. The smooth hydrophobic (HPo) tube for the dropwise condensation is obtained by depositing the SAMs on the plasma-cleaned Bare tube.

Advancing *θ*_a_, static *θ*_s_ and receding contact angles *θ*_r_ for the tested surfaces are follow. Bare: *θ*_a_ < 10°, *θ*_s_ < 10°, and *θ*_r_ = 0°. HPo: *θ*_a_ = 123.2 ± 2.7°, *θ*_s_ = 111.2 ± 0.8°, and *θ*_r_ = 90.7 ± 2.7°. SHPo: *θ*_a_ = 168.2 ± 2.9°, *θ*_s_ = 167.0 ± 2.1°, and *θ*_r_ = 165.9 ± 2.1°. LIS: *θ*_a_ = 111.9 ± 1.7°, *θ*_s_ = 110.0 ± 1.3°, and *θ*_r_ = 108.2 ± 1.4°.

### Surface characterization

Advancing, static and receding contact angles for all samples are measured and analyzed using a contact angle analyzer SDS-TEZD from FEMTOFAB. All SEM images are obtained on a Carl Zeiss MERLIN at an imaging voltage of 10 kV. The ESEM (environmental scanning electron microscope) experiments are conducted in an ESEM chamber (Philips XL30 ESEM FEG). The test samples are fixed on a cold stage at the surface temperature *T*_s_ of ~2 °C, and the chamber environment is controlled as the vapor pressure *P*_vacuum_ of ~720 Pa and *S* of ~1.01.

### Rotation system

The motor system to rotate the test tube is composed of a DC motor and a motor speed controller. The DC motor is connected to a reduction gear and can rotate the test tube at 2‒8 RPM. The speed controller is made with a circuit board. A power supply is connected to the controller and consumes 10 V and 0.02 A for 2 RPM of the test tube.

### Condensation experimental apparatus

All condensation experiments are conducted under well-controlled environmental conditions, shown in the Supplementary Note 11. Non-condensable gases, which significantly deteriorate the condensation heat transfer process by acting as a diffusion barrier for water vapor at the droplet interface^43^, are carefully controlled by using a vacuum pump. A stainless steel frame of this chamber is wrapped by resistive silicone rubber heater lines and then is insulated. The temperature of these heater lines is controlled by a slidac transformer (maximum capacity of 5 kW and output voltage of 0–240 V) to maintain the constant chamber wall temperature. A KF flanged bellow connected to an angle valve is integrated on the top of the chamber for a vacuum pump (BT-35A oil rotary vacuum pump, BESTECH). A liquid nitrogen cold trap is connected between the bellow line and the vacuum pump to block the inflow of moisture to the vacuum pump. A pressure gauge (275 Convectron Pirani Vacuum Gauge, MKS) is used to monitor vacuum pressure in the chamber.

Cooling water from a thermal bath (maximum flow rate of 40 L/min, maximum pump pressure of 3.3 bar, cooling capacity of 0.9 kW at 0 °C and temperature accuracy of ± 0.1 °C at 15 °C, Lab Companion, Korea) is circulated along two insulated water flow lines which are fed into the chamber. A flow meter (FLEX-HD1K, Honsberg Instruments, Germany) is integrated to monitor the flow rate.

Degassed water vapor is introduced into the chamber from a stainless steel water reservoir. This reservoir is wrapped by a band heater and its temperature was controlled by a heater controller (TZ4L, Autonics). Two needle valves are connected to two stainless steel tubes incorporated with a cover of the reservoir. The first tube is used to fill DI water into the reservoir. This tube line connects the bottom of the reservoir to the ambient. The second tube is used to supply degassed water vapor into the chamber. This tube line connects the top of the reservoir to the inside of the chamber, and it is wrapped by a heating tape.

Tube inlet and outlet temperatures are measured by two thermocouple probes. A saturation vapor temperature in the chamber is measured by a thermocouple probe wrapped with a wet bulb wick. All the thermocouples are calibrated before the experiment. The temperatures and the pressure information are monitored by using DAQ system. Condensation behavior is recorded by a CCD camera.

### Condensation experimental procedure

The resistive silicone rubber heater lines wrapping the chamber are first turned on to heat up the chamber wall. The water reservoir is fully filled with DI water using a syringe line through the needle valve connecting to the first stainless steel tube, and then the needle valve is closed. Subsequently, the needle valve integrated with the second tube is fully opened, and the heater controller connected to the band heater is turned on. The DI water in the reservoir is boiled at 300 °C for 40 min to thoroughly remove any non-condensable gases in the water and the reservoir. During this boiling process, a stainless bath is placed below the inlet of the inflow line to collect any leaving water. After the degassing process, the heater controller is set to 100 °C, all needle valves are closed and the vapor inflow line is integrated into the chamber.

The test tube is integrated with two bellows tubes which connect to the two insulated water flow lines, and then it is positioned in the center of the chamber using the two supporters. The thermocouple probe wrapped with a wet bulb wick is placed below the tube. Then the door of the chamber is closed.

Next, the cold trap is filled with liquid nitrogen and the vacuum pump is turned on. When the target vacuum pressure is achieved, cold water of 5 L/min is circulated from the thermal bath. Then the needle valve of the second tube of the reservoir is opened to introduce degassed water vapor into the chamber, and simultaneously the vacuum pump line is closed. Once at steady state, the temperature, pressure and flow rate data are measured by using the DAQ system, and the condensation behavior is recorded by a CCD camera.

### Contact angle measurement in a water environment

We experimentally measured the contact angle for the Krytox 1506 oil droplet on a silane-functionalized Cu surface in a water environment (*θ*_o/w_ = ~28.9°), as shown in Fig. S10. Figure S10A displays the experimental procedure to measure the contact angle. Krytox oil is denser than water; hence, we placed the surface on the bottom of the glass box filled with distilled water, lightly sit the oil droplet on the surface, and then measured the contact angle using a CCD camera. Figure S10B shows a photograph of the measured contact angle.

### Oil thickness measurement

We measured *δ*_o_, the total thickness of the impregnated oil on the surface, by the difference in the mass of the tube before and after oil impregnation using a precision electronic balance (Max. capacity of 220 g, readability of 0.1 mg, repeatability of 0.1 mg and linearity of ± 0.2 mg, AS 220.R1, RADWAG). Before applying the oil, the silane-functionalized CuO nanostructured copper with stainless fittings showed the average mass of ~134.1391 g. When the oil was applied to this tube, the average mass was found to be ~134.1451 g. The average mass of the impregnated oil was obtained by the mass difference before and after oil applying, such as 134.1451 g - 134.1391 g = ~6 mg. The total volume of the impregnated oil was achieved by 6 mg × 1880 kg/m^3^ = 3.1915 × 10^-9^ m^3^. *δ*_o_ was calculated by dividing the volume of the impregnated oil by the surface area of the tube, such as 3.1915 × 10^-9^ m^3^/(π × 0.006 m × 0.13 m) = 3.1915 × 10^-9^ m^3^/2.4504 × 10^-3^ m^2^ = ~1.3 μm.

## Supplementary information

Supplementary Movie S1

Supplementary Movie S2

Supplementary Movie S3

Supplementary Movie S4

Supplementary Movie S5

Supplementary Information
