# Supplementary material for: Brushed lubricant-impregnated surfaces (BLIS) for long-lasting high condensation heat transfer
Source: Sci Rep. 2020 Feb 19;10:2959. doi: 10.1038/s41598-020-59683-z (PMC7031390; doi:10.1038/s41598-020-59683-z)
Supplement: Supplementary file 6 — Supplementary Information [file 41598_2020_59683_MOESM6_ESM.pdf]

Supplementary Information

Brushed lubricant-impregnated surfaces (BLIS) for long-lasting high condensation heat transfer

Donghyun Seo, Jaehwan Shim, Choongyeop Lee*, and Youngsuk Nam*

Department of Mechanical Engineering, Kyung Hee University, Yongin 446-701, Korea
*cylee@khu.ac.kr, *ysnam1@khu.ac.kr

Contents

Supplementary Note 1. **Descriptions of movies S1 to S5**

Supplementary Note 2. **Droplet wetting morphology during oil depletion**

Supplementary Note 3. **Brushing system**

Supplementary Note 4. **Robustness of CuO nanostructures during brushing**

Supplementary Note 5. **RPM effect**

Supplementary Note 6. **Calculation for condensation heat transfer coefficient**

Supplementary Note 7. **Heat transfer model**

Supplementary Note 8. Droplet removal size characterization

Supplementary Note 9. **Ideas for using BLIS**

Supplementary Note 10. **CuO SEM images**

Supplementary Note 11. **Condensation experimental setup**

Supplementary Note 12. Contact angle measurement in a water environment

Supplementary Note 13. The lifetime for LIS

Supplementary Note 1

Descriptions of movies S1 to S5

Movie S1 shows the effect of the oil replenishment by the oil brushing system. Before the rotation, BLIS undergoes rapid oil depletion and condensation performance degradation, like LIS at S = ~1.5. When the tube begins to rotate at 2 RPM, the lost oil is quickly replenished.

Movie S2 shows that dropwise condensation on HPo gradually turns into a water film at S = ~1.5 due to the coating degradation.

Movie S3 shows that dropwise condensation on SHPo gradually turns into a water film at S = ~1.5 due to the surface flooding phenomenon.

Movie S4 shows that dropwise condensation on LIS gradually turns into a water film at S = ~1.5 due to the rapid oil depletion.

Movie S5 shows the stable and long-term dropwise condensation on BLIS at S = ~1.5. The tube rotates at 2 RPM.

Supplementary Note 2

Droplet wetting morphology during oil depletion

To demonstrate the changes in the droplet wetting morphology during oil depletion, we calculate the modified roughness factor. Figures S1A and S1B show the geometry of a single CuO nanostructure where the lubricant oil is depleted as much as depletion fraction ϕ_d. ϕ_d = 1 or 0 indicates that the oil within the nanostructure fills as much as the height of the structure or is completely removed. When we assume the shape of the CuO nanostructure as a very thin triangle, the total surface area of the CuO nanostructure A_{CuO} can be expressed as,

$$A_{CuO} = hb/2 = h\left(h\tan\left(\alpha/2\right)\right) = h^2\tan\left(\alpha/2\right), \text{ (S1)}$$

where h is the height of the CuO nanostructure (~1 μm), b is the bottom length of the CuO nanostructure and α is the shape angle of the CuO nanostructure. The surface area of the exposed CuO nanostructure after depletion $A_{CuO,d}$ can be expressed as,

$$A_{CuO,d} = \phi_d h b_d/2 = \phi_d h\left(\phi_d h\tan\left(\alpha/2\right)\right) = \phi_d^2 h^2\tan\left(\alpha/2\right), \text{ (S2)}$$

where b_d is the bottom length of the exposed CuO nanostructure. When we assume that the surface roughness factor is proportional to the surface area of the CuO nanostructures, the roughness factor r can be expressed as,

$$r \cong A_{CuO} = h^2\tan\left(\alpha/2\right). \text{ (S3)}$$

Then the modified roughness factor r_d, which is indicate of the surface roughness factor

when the impregnated lubricant is depleted as much as ϕ_d, can be expressed as,

$$r_d \cong A_{CuO,d} = \phi_d^2 h^2 \tan(\alpha / 2) = \phi_d^2 r \text{ . (S4)}$$

Then the switch of the wetting morphology between stage 2 and 3 can be estimated by the normalized dimensionless energy criteria E^*, which is expressed as $E^* = -1 / (r\cos\theta_a)$[1]. This criterion is based on the comparison between wetting energies for homogeneous wetting ($\cos\theta_a^W = r\cos\theta_a$, where θ_a^W is the advancing angle of a Wenzel state droplet.) and non-wetting ($\cos\theta_a^C = -1$, where θ_a^C is the advancing angle of a Cassie state droplet.). When $E^* < 1$, the droplet tries to minimize the contact with the surface by growing over nanostructures rather than spreading in between nanostructures. We introduce the change in the lubricant thickness into the dimensionless energy criteria by replacing r with the modified roughness factor $r_d = \phi_d^2 r$ like the following,

$$E_d^* = \frac{-1}{\phi_d^2 r \cos\theta_a} \text{ . (S5)}$$

On the CuO nanostructures used in this study, the critical depletion fraction $\phi_{d,crit}$, where the droplet wetting morphology is changed, is estimated to be ~0.67. As the result, the droplet morphology is expected to prefer the hemispherical morphology in stage 2 and the spherical morphology in stage 3 when $\phi_d < 0.67$ ($E_d^* > 1$) and $\phi_d > 0.67$ ($E_d^* < 1$), respectively, as shown in Fig. S1C.

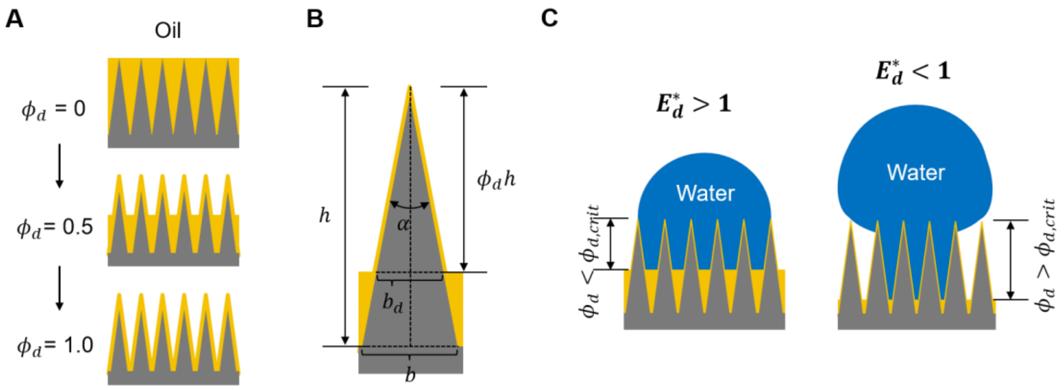

Figure S1. Droplet wetting morphology during oil depletion. (**A**, **B**) Schematic illustration of the oil depletion within CuO nanostructures. (**A**) ϕ_d is the depletion fraction. ϕ_d = 1 or 0 indicates that the oil within the nanostructure fills as much as the height of the structure or is completely removed. (**C**) Predicted droplet wetting morphology depending on ϕ_d. When $\phi_d < \phi_{d,crit}$ ($E_d^* > 1$), the hemispherical morphology in stage 2 is expected to be thermodynamically preferred. When $\phi_d > \phi_{d,crit}$ ($E_d^* < 1$), the spherical morphology in stage 3 is predicted.

Supplementary Note 3

Brushing system

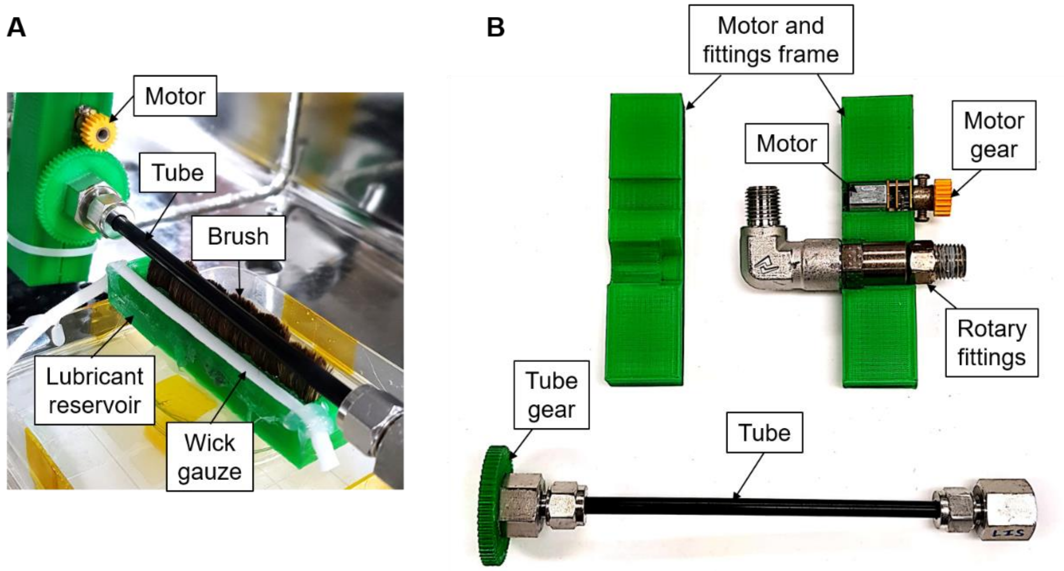

Figure S2. (**A**, **B**) Brushing system. (**A**) A photograph of the brushing system integrated with a test tube inside the condensation chamber. The brush is attached to the wall of the lubricant reservoir. The reservoir is positioned in consideration of the droplet drainage direction to prevent the swept droplets to be drained out without collecting inside the reservoir. A wick gauze is placed at the front of the brush so that falling droplets along the brush can be quickly removed. (**B**) A photograph of each component consisting of the brushing system. The frame and gear are made with a 3D printer.

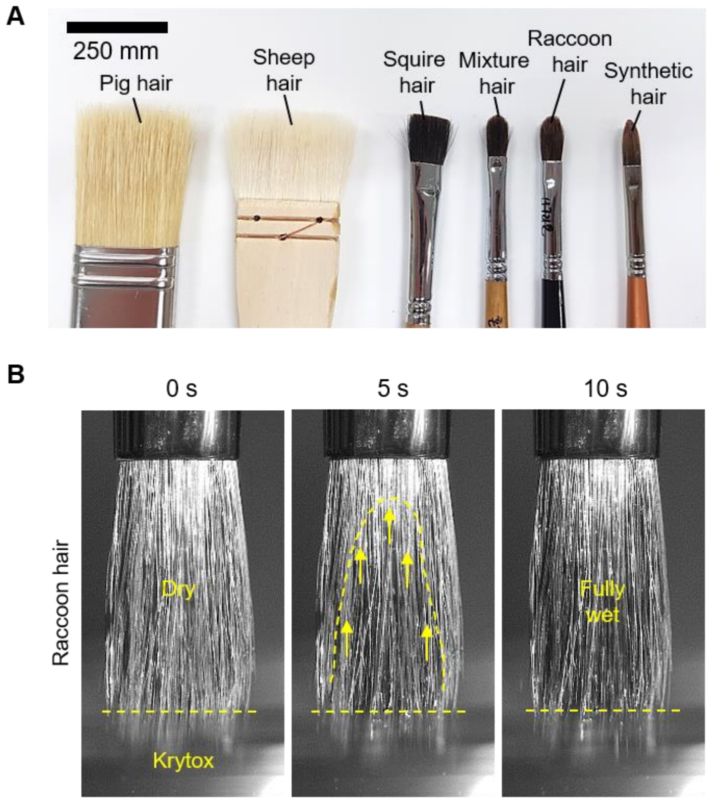

Figure S3. (**A**) Tested oil painting brushes. Natural animals (pig, sheep, squire and raccoon), mixture and synthetic hairs are investigated. (**B**) As soon as all oil painting brushes touch Krytox 1506, the oil sucks up quickly. The difference in oil wicking performance among the tested brushes is negligible; hence, we chose the softest brush, the mixture hair (Z-8250, Herend), for the brushing system. The brush hairs have a height of ~15.5 mm and the shape is filbert. We squeezed the head of the brush so that a lot of hair can contact the tube surface. The quantitative capillary force could not be calculated since many hairs are irregularly distributed, but the dry brush can pull the oil from the bottom to the tip in about 10 s. When using the brush for over 48 h condensation experiments, we did not observe any dry out of the tube surface.

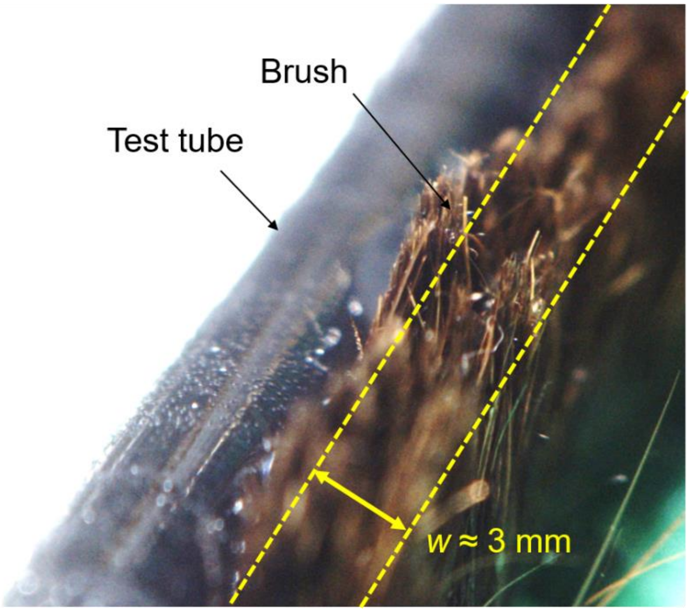

Figure S4. A test tube and a brush. The continuous oil replenishment through the physical contact of the brush to the tube surface will lead to the oil depletion on the brush. However, a thick brush with a width of half the tube diameter ($D_{o.d.}$ = ~6 mm) can quickly pull oil from the lubricant reservoir and store it inside, which can prevent the brush to be wet with water. This fact can be inferred through the observation that the brush sustainably wet with oil even for over 48 hours' condensation under high supersaturations.

Supplementary Note 4

Robustness of CuO nanostructures during brushing

To prevent damage to CuO nanostructures due caused by continuous brushing, we used a very soft brush and it was placed in light contact with the surface. In addition, the brush is coated with lubricant oil, which can minimize the abrasion between the brush and the

surface. Figure S5 shows SEM images of CuO nanostructures before and after the brushing tests. Even after 48 hours, the CuO nanostructures were not damaged.

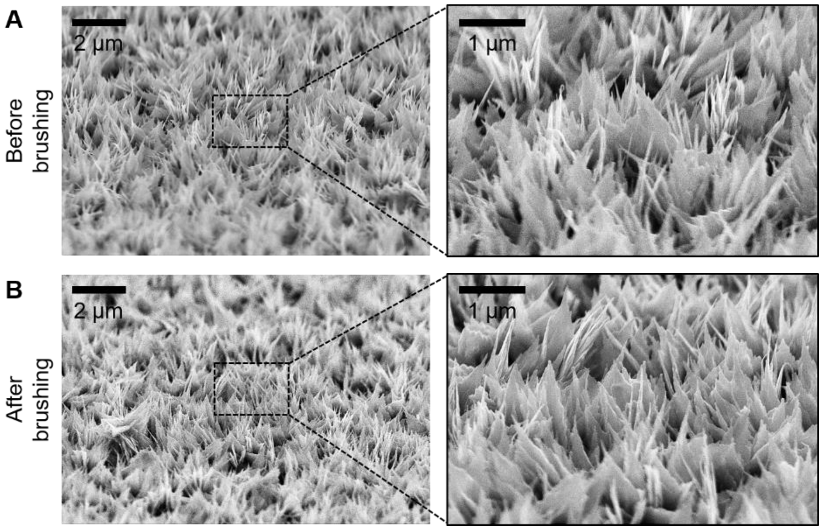

Figure S5. (**A**, **B**) SEM images of CuO nanostructures (**A**) before and (**B**) after brushing. Before taking the images, all the infused oil within nanostructures was removed using the electron beam energy of SEM.

Supplementary Note 5

RPM effect

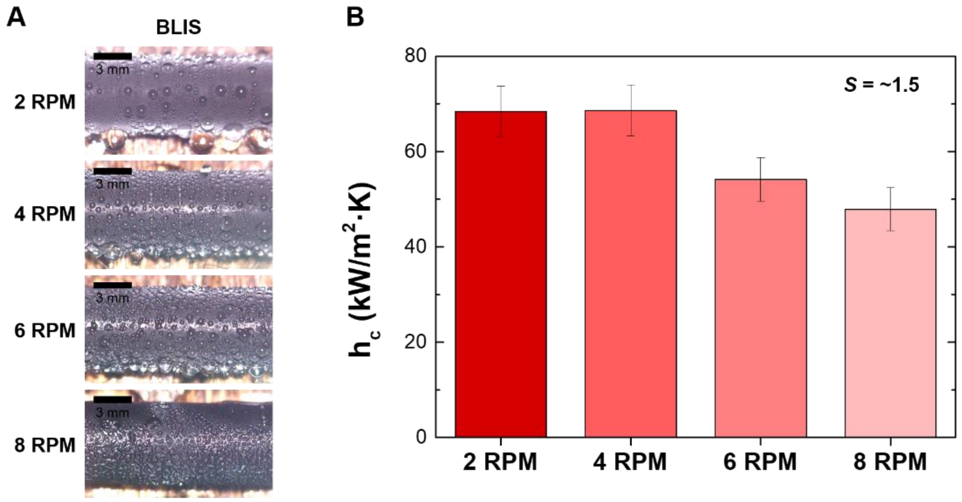

Figure S6. RPM effect. (**A**) Steady-state condensation images of BLIS depending on RPM. The increase in RPM causes over-application of oil on the tube wall. The droplet packing phenomenon begins to observe at 6 RPM and almost all droplets are packed at 8 RPM. (**B**) h_c as a function of RPM at S of ~1.5. From 6 RPM, h_c gradually reduces due to the increased lubricant thickness and the droplet packing configuration which interrupts the droplet coalescence and growth.

Supplementary Note 6

Calculation for condensation heat transfer coefficient

To calculate the condensation heat transfer coefficient h_c, the overall heat transfer coefficient U which indicates the heat transfer performance between surrounding vapor and the cold water inside the test tube is first calculated as follow,

$$U = \frac{q''}{\Delta T_{LMTD}} = \frac{\dot{m} c_{p,l} \left(T_{out} - T_{in} \right)}{A_o \Delta T_{LMTD}}. \text{ (S6)}$$

Here, q'' is the heat flux through the tube surface, $\dot{m}$ is the mass flow rate of the cooling water, $c_{\mathrm{p,l}}$ is the specific heat of cooling water, T_{out} is the tube outlet temperature, T_{in} is the tube inlet temperature, A_{o} is the tube outer surface area (= $\pi d_{\mathrm{o}}L$, where d_{o} is the tube outer diameter and L is the tube length.) and ΔT_{LMTD} is the logarithmic mean temperature difference (LMTD) which is defined as,

$$\Delta T_{LMTD} = \frac{\left(T_v - T_{in} \right) - \left(T_v - T_{out} \right)}{\ln \left(\dfrac{T_v - T_{in}}{T_v - T_{out}} \right)}, \text{ (S7)}$$

where T_{v} is the vapor saturation temperature which is measured by wet-bulb thermocouples inside the condensation chamber. The h_{c} which represents the heat transfer performance from surrounding vapor to the tube surface is calculated by excluding the effects of the internal cooling water flow and conduction through the tube as follow,

$$h_c = \left(\frac{1}{U} - \frac{A_o}{A_i h_i} - \frac{A_o \ln \left(d_o / d_i \right)}{2\pi L k_{Cu}} \right)^{-1}, \text{ (S8)}$$

where A_{i} is the internal tube surface area (= $\pi d_{\mathrm{i}}L$, where d_{i} is the tube inner diameter), k_{Cu} is the Cu thermal conductivity and h_{i} is heat transfer coefficient of the internal cooling water flow expressed as,

$$h_i = \frac{k_l}{d_i}\frac{(f/8)(\mathrm{Re}-1000)\mathrm{Pr}}{1+12.7(f/8)^{1/2}\left(\mathrm{Pr}^{2/3}-1\right)}, \text{ (S9)}$$

where k_l is the thermal conductivity of the cooling water, f is the pipe friction factor (= $\left(0.79\ln\mathrm{Re}-16.4\right)^{-2}$), Re is Reynolds number of the cooling water flow (= $\rho_\mathrm{l} v d_\mathrm{i} / \mu_\mathrm{l}$, where ρ_l is the cooling water density, v is the cooling water flow velocity and μ_l is the dynamic viscosity of the cooling water) and Pr is the Prandtl number. The condensation heat flux q_c'' can be calculated as,

$$q_c'' = h_c \Delta T_{LMTD}. \text{ (S10)}$$

Supplementary Note 7

Heat transfer model

We calculate the condensation heat transfer coefficient h_c using the heat transfer model that incorporates the thermal resistance model for droplet growth, the emergent droplet wetting morphology, and droplet distribution theory[2-5]. Note that in the calculation the surrounding saturated vapor temperature T_v and surface temperature T_s are assumed to be constant, and the marangoni effect inside a droplet is ignored. First, the temperature drop by droplet curvature ΔT_c is given by

$$\Delta T_c = \frac{R_{\min}}{R}\left(T_v - T_s\right) = \frac{2T_v\gamma}{R h_{hg}\rho_w}, \text{ (S11)}$$

where R_{min} is the minimum radius for droplet nucleation expressed as $R_{min} = 2T_{sat}\gamma / h_{fg}\rho_w\Delta T$ and R is the droplet radius. Here, γ is the water-vapor interfacial tension, h_{fg} is the latent heat of vaporization and ρ_w is the condensate density. The temperature drop by vapor-liquid interface ΔT_{int} is given by

$$\Delta T_{\text{int}} = \frac{q}{h_{\text{int}} 2\pi R^2 \left(1 - \cos\theta_a\right)}, \text{ (12)}$$

where q is the heat transfer rate through the individual droplet and h_{int} is the interfacial heat transfer coefficient which is given by

$$h_{\text{int}} = \frac{2\alpha}{2-\alpha} \frac{1}{\sqrt{2\pi R_g T_s}} \frac{h_{fg}^2}{v_g T_s}, \text{ (S13)}$$

where α is the condensation coefficient, R_g is the specific gas constant (= ~461.5 J/kg·K) and v_g is the water vapor specific volume. The temperature drop by conduction through the droplet ΔT_d is given by

$$\Delta T_d = \frac{q\theta_a}{4\pi R k_w \sin\theta_a}, \text{ (S14)}$$

where k_w is the condensate thermal conductivity. The temperature drop by the hydrophobic

SAM coating layer ΔT_{hc} is given by

$$\Delta T_{hc} = \frac{q\delta_{hc}}{f_s \pi R^2 k_{hc} \sin^2\theta_a}, \text{ (S15)}$$

where δ_{hc} is the coating layer thickness (~10 nm), ϕ_{eff} is the effective solid fraction of the nanostructures and k_{hc} is the thermal conductivity of the SAM coating layer (~0.2 W/m·K). The temperature drop by a parallel heat transfer pathway from the base of the droplets to the substrate surface ΔT_{ns} is given by

$$\Delta T_{ns} = \frac{q}{\pi R^2 k_{hc} \sin^2\theta_a}\left[\frac{k_{ns} f_s}{\delta_{hc}k_{ns} + hk_{hc}} + \frac{k_w(1-f_s)}{\delta_{hc}k_w + hk_{hc}}\right]^{-1}, \text{ (S16)}$$

where k_{ns} is the thermal conductivity of nanostructures and k_{o} is the thermal conductivity of the impregnated oil. Incorporating the all temperature drops, the heat transfer rate through the single droplet is given by

$$q(R,\theta_a) = \frac{\pi R^2\left(\Delta T - \dfrac{2T_v\gamma}{Rh_{fg}\rho_w}\right)}{\dfrac{1}{2h_{\mathrm{int}}(1-\cos\theta_a)} + \dfrac{R\theta_a}{4k_w \sin\theta_a} + \dfrac{1}{k_{hc}\sin^2\theta_a}\left[\dfrac{k_{nc} f_s}{\delta_{hc}k_{ns} + hk_{hc}} + \dfrac{k_o(1-f_s)}{\delta_{hc}k_o + hk_{hc}}\right]^{-1}},$$

(S17)

where ΔT is the surface subcooling temperature (= $T_{\mathrm{v}} - T_{\mathrm{s}}$). The surface heat flux q'' can be calculated by combining the above heat transfer rate through the single droplet with the

droplet size distribution as follow,

$$q'' = \int_{R_{\min}}^{R_e} q(R)n(R)dR + \int_{R_e}^{\hat{R}} q(R)N(R)dR \text{ . (S18)}$$

Here, $n(R)$ and $N(R)$ are the droplet size distribution for small droplets of $R_{\min} < R < R_e$ and large droplets of $R_e < R < \hat{R}$, respectively. The R_e is the radius when droplets growing by direct vapor addition begins to coalesce and grow by coalescence, and it is given by $R_e = \langle L \rangle / 2 = 1 / (16n)^{0.5}$. Then the $n(R)$ and $N(R)$ are given by

$$n(R) = \frac{1}{3\pi R_e^3 \hat{R}} \left(\frac{R_e}{\hat{R}} \right)^{-2/3} \frac{R(R_e - R_{\min})}{R - R_{\min}} \frac{A_2 R + A_3}{A_2 R_e + A_3} \exp(B_1 + B_2) \text{, (S19)}$$

$$N(R) = \frac{1}{3\pi R^2 \hat{R}} \left(\frac{R}{\hat{R}} \right)^{-2/3} \text{, (S20)}$$

Where

$$B_1 = \frac{A_2}{\tau A_1} \left[\frac{R_e^2 - R^2}{2} + R_{\min}(R_e - R) - R_{\min}^2 \ln\left(\frac{R - R_{\min}}{R_e - R_{\min}} \right) \right] \text{, (S21)}$$

$$B_2 = \frac{A_3}{\tau A_1} \left[R_e - R - R_{\min} \ln\left(\frac{R - R_{\min}}{R_e - R_{\min}} \right) \right] \text{, (S22)}$$

$$\tau = \frac{3R_e^2\left(A_2R_e + A_3\right)^2}{A_1\left(11A_2R_e^2 - 14A_2R_eR_{\min} + 8A_3R_e - 11A_3R_{\min}\right)} \text{, (S23)}$$

$$A_1 = \frac{\Delta T}{h_{fg}\rho_w\left(1-\cos\theta_a\right)^2\left(2+\cos\right)\theta_a} \text{, (S24)}$$

$$A_2 = \frac{\theta_a}{4k_w\sin\theta_a} \text{, (S25)}$$

$$A_3 = \frac{1}{2h_{\text{int}}\left(1-\cos\theta_a\right)} + \frac{1}{k_w\sin^2\theta_a}\left[\frac{k_{ns}f_s}{\delta_{hc}k_{ns} + hk_{hc}} + \frac{k_o\left(1-f_s\right)}{\delta_{hc}k_o + hk_{hc}}\right]^{-1} \text{. (S26)}$$

For FWC of Bare, the classical Nusselt model[6] is used as follow,

$$h_c = 0.729\left[\frac{g\rho_w\left(\rho_w - \rho_v\right)k_w^3h'_{fg}}{\mu_wD_o\Delta T}\right]^{1/4} \text{, (S27)}$$

where ρ_v is the water vapor density and h'_{fg} is the modified latent heat of vaporization that is given by,

$$h'_{fg} = h_{fg} + 0.68c_{p,w}\Delta T \text{, (S28)}$$

where $c_{p,w}$ is the condensate specific heat.

Supplementary Note 8

Droplet removal size characterization

Table S1. Average droplet removal diameter $2\hat{R}$.

	Average $2\hat{R}$	Standard deviation
HPo (1.0 < *S* < 1.6)	2.68 mm	0.38 mm
SHPo (*S* = ~1.08, jumping)	~15 μm[7]	-
SHPo (*S* = ~1.19, flooding)	2.32 mm	0.31 mm
SHPo (*S* > 1.19, flooding)	-	-
BLIS (1.0 < *S* < 1.6)	0.88 mm	0.06 mm

Supplementary Note 9

Ideas for using BLIS

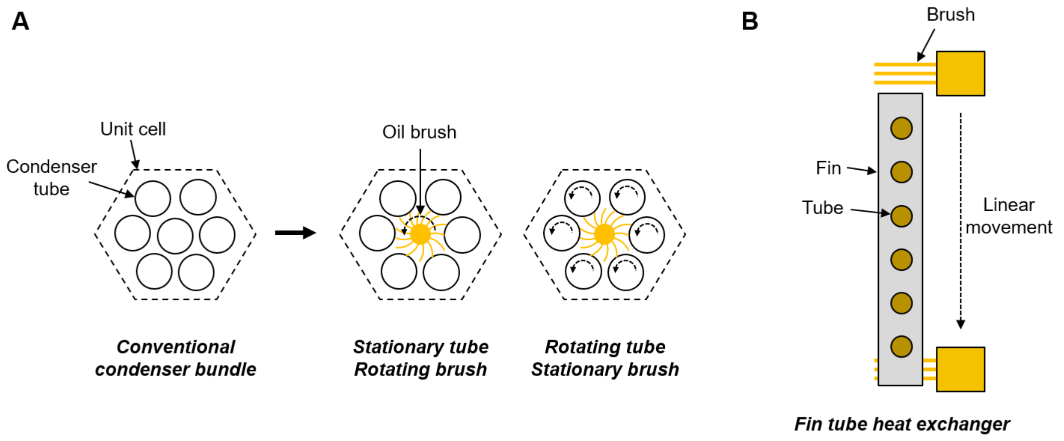

Figure S7. Schematic showing examples of a brush applied to (**A**) shell and tube condenser bundle and (**B**) fin-tube heat exchangers. (**A**) BLIS can be utilized by replacing

a tube at the center of the hexagonal bundle to a brush. (**B**) The brush can be connected to a system that moves up and down linearly so that lost oil can be replenished and condensing droplets can be swept.

Supplementary Note 10

CuO SEM images

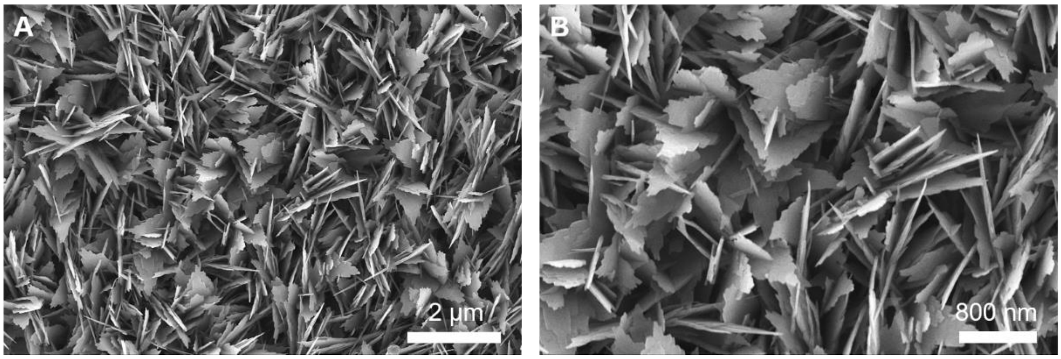

Figure S8. SEM images of tested CuO nanostructures. (**A**, **B**) Knife-like CuO nanostructures have height h = ~1.0 μm, pitch p = ~300 nm, solid fraction f_s = ~0.023, roughness factor r = ~10.2.

Supplementary Note 11

Condensation experimental setup

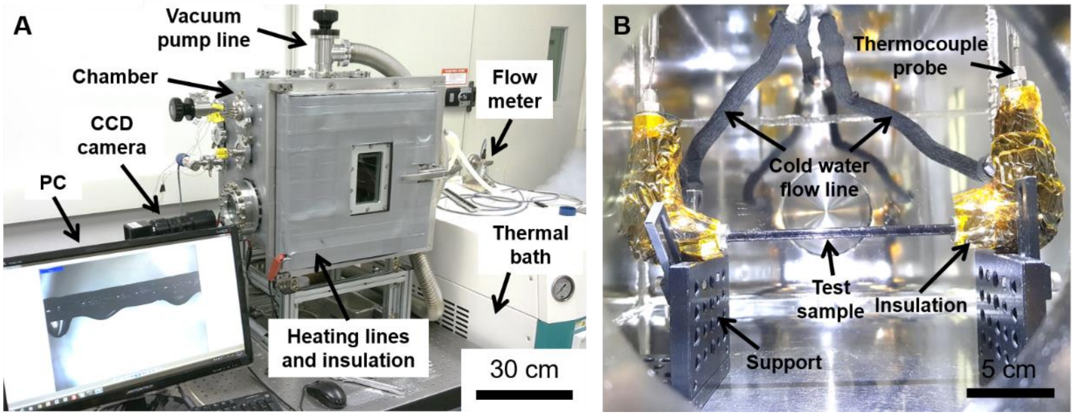

Figure S9. Condensation experimental setup. (**A**, **B**) Condensation setup. Non-condensable gases (NCGs) are limited using a vacuum pump, and a wide range of condensation rate conditions (10 kW/m^2 < q_c" < 850 kW/m^2) is accurately controlled. Cooling water from the thermal bath is circulated inside the test tube in the chamber, and degassed vapor is supplied from the water reservoir located behind the chamber. The q_c" is calculated by measuring inlet T_{in}, outlet T_{out}, vapor saturation temperatures T_v and mass flow rate $\dot{m}$ of cooling water. The oil depletion dynamics are observed using a CCD camera.

Supplementary Note 12

Contact angle measurement in a water environment

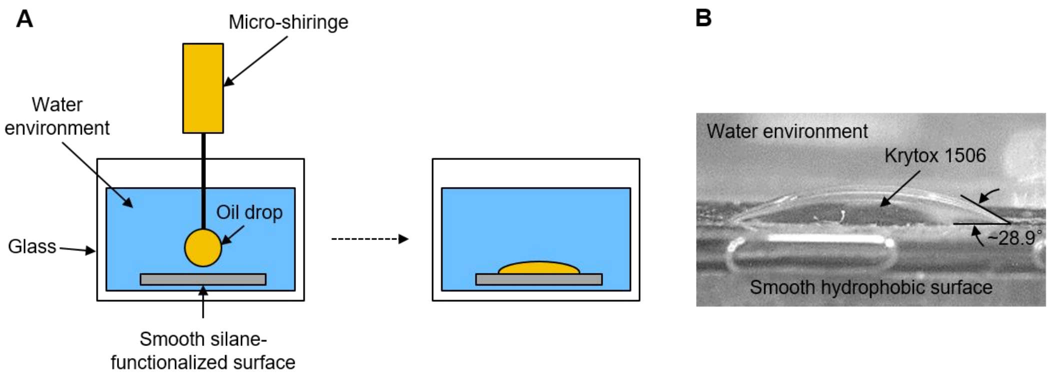

Figure S10. (**A**) Schematic and of an experimental procedure to measure the intrinsic contact angle for a droplet on a solid surface in a water environment $\theta_{o/w}$. (**B**) A photograph showing $\theta_{o/w}$.

Supplementary Note 13

The lifetime for LIS

The lifetime for LIS defined as t_{stage1} seems very short (Figure 2E). We understand the reason as the criteria for evaluating the lifetime for LIS. In the manuscript, we defined the lifetime for LIS as t_{stage1} when the sliding droplet size is maintained to be about $2R_{max}$ = ~1.2 mm. If the lifetime for LIS is set for $2R_{max}$ = 2 mm, the lasting time for LIS can be over twice as long. As such, the lifetime for LIS can be different according to the criteria for the lifetime.

Second, as we reported in the manuscript, the oil depletion rate is very sensitive to the condensation rate. The fast oil depletion of t_{stage1} < 3 min is only for very harsh condensation conditions where non-condensable gases (NCGs) are removed. If LIS was

tested under NCGs, stage 1 can last for tens of minutes. If the criteria for $2R_{max}$ = 2 mm is considered here, LIS can last more than one hour under NCGs.

Third, the lifetime for LIS highly depends on surface structures[8], lubricant properties such as interfacial tension and viscosity[9], and lubricant thickness. Krytox 1506 used in our work has relatively small kinematic viscosity of ~62 mm^2/s. And the thickness of the applied oil was maintained thinly by removing excess oil on the surface so that the conduction resistance across the impregnated lubricant layer was minimized. Smaller and denser structures, higher viscosity and thicker oils can show a longer lifetime than our results.

Supplementary References

1 Enright, R., Miljkovic, N., Al-Obeidi, A., Thompson, C. V. & Wang, E. N. Condensation on Superhydrophobic Surfaces: The Role of Local Energy Barriers and Structure Length Scale. *Langmuir* **28**, 14424-14432, doi:10.1021/la302599n (2012).

2 Miljkovic, N., Enright, R. & Wang, E. N. Modeling and Optimization of Superhydrophobic Condensation. *J. Heat Transfer* **135**, 111004-111004-111014, doi:10.1115/1.4024597 (2013).

3 Kim, H. & Nam, Y. Condensation behaviors and resulting heat transfer performance of nano-engineered copper surfaces. *Int. J. Heat Mass Transf.* **93**, 286-292, doi:https://doi.org/10.1016/j.ijheatmasstransfer.2015.09.079 (2016).

4 Preston, D. J. *et al.* Heat Transfer Enhancement During Water and Hydrocarbon Condensation on Lubricant Infused Surfaces. *Sci. Rep.* **8**, 540, doi:10.1038/s41598-017-18955-x (2018).

5 Seo, D. *et al.* Passive Anti-flooding Superhydrophobic Surfaces. *ACS Appl. Mater. Interfaces*, doi:10.1021/acsami.9b17943 (2019).

6 Carey, V. P. *Liquid Vapor Phase Change Phenomena: An Introduction to the Thermophysics of Vaporization and Condensation Processes in Heat Transfer Equipment, Second Edition*. (Taylor & Francis, 2007).

7 Miljkovic, N. *et al.* Jumping-Droplet-Enhanced Condensation on Scalable Superhydrophobic Nanostructured Surfaces. *Nano Lett.* **13**, 179-187, doi:10.1021/nl303835d (2013).

8 Kim, P., Kreder, M. J., Alvarenga, J. & Aizenberg, J. Hierarchical or Not? Effect of the Length Scale and Hierarchy of the Surface Roughness on Omniphobicity of Lubricant-Infused Substrates. *Nano Lett.* **13**, 1793-1799, doi:10.1021/nl4003969

(2013).

9 Liu, Y., Wexler, J. S., Schönecker, C. & Stone, H. A. Effect of viscosity ratio on the shear-driven failure of liquid-infused surfaces. *Physical Review Fluids* **1**, 074003, doi:10.1103/PhysRevFluids.1.074003 (2016).
